# In Vitro and In Vivo Metabolomic Profiling after Infection with Virulent Newcastle Disease Virus

**DOI:** 10.3390/v11100962

**Published:** 2019-10-18

**Authors:** Panrao Liu, Yuncong Yin, Yabin Gong, Xusheng Qiu, Yingjie Sun, Lei Tan, Cuiping Song, Weiwei Liu, Ying Liao, Chunchun Meng, Chan Ding

**Affiliations:** 1 Shanghai Veterinary Research Institute, Chinese Academy of Agricultural Sciences, Shanghai 200241, China; liupanrao@163.com (P.L.); gongyabin2018@163.com (Y.G.); xsqiu1981@shvri.ac.cn (X.Q.); sunyingjie@shvri.ac.cn (Y.S.); tanlei@shvri.ac.cn (L.T.); scp@shvri.ac.cn (C.S.); liuweiwei@shvri.ac.cn (W.L.); liaoying@shvri.ac.cn (Y.L.); 2 College of Veterinary Medicine, Yangzhou University, Yangzhou 225009, Jiangsu, China; yzyuncong@hotmail.com; 3 Jiangsu Co-innovation Center for Prevention and Control of Important Animal Infectious Diseases and Zoonoses, Yangzhou 225009, China

**Keywords:** virulent Newcastle disease virus, metabolomic analysis, in vitro and in vivo, UHPLC-QTOF-MS

## Abstract

Newcastle disease (ND) is an acute, febrile, highly contagious disease caused by the virulent Newcastle disease virus (vNDV). The disease causes serious economic losses to the poultry industry. However, the metabolic changes caused by vNDV infection remain unclear. The objective of this study was to determine the metabolomic profiling after infection with vNDV. DF-1 cells infected with the vNDV strain Herts/33 and the lungs from Herts/33-infected specific pathogen-free (SPF) chickens were analyzed via ultra-high-performance liquid chromatography/quadrupole time-of-flight tandem mass spectrometry (UHPLC-QTOF-MS) in combination with multivariate statistical analysis. A total of 305 metabolites were found to have changed significantly after Herts/33 infection, and most of them belong to the amino acid and nucleotide metabolic pathway. It is suggested that the increased pools of amino acids and nucleotides may benefit viral protein synthesis and genome amplification to promote NDV infection. Similar results were also confirmed in vivo. Identification of these metabolites will provide information to further understand the mechanism of vNDV replication and pathogenesis.

## 1. Introduction

Newcastle disease (ND) is an acute, febrile, and highly contagious disease caused by velogenic Newcastle disease virus (vNDV). It is found worldwide. It mainly affects poultry, such as chickens, pheasants, and turkeys, in which it has high incidence and high lethality. It is listed as a notifiable disease by the OIE-World Organization for Animal Health [1]. NDV strains can be categorized into three main pathotypes: Lentogenic, mesogenic, and velogenic, depending on the severity of the disease produced in chickens. Velogenic NDV causes a serious respiratory and neurological disease in all species of birds and is an economically important infectious agent, causing substantial losses to the poultry industry [2,3]. NDV is an enveloped, single-stranded, non-segmented, negative-sense RNA virus of the Paramyxoviridae family. Although significant progress has been made in research into the pathogenesis of vNDV, the intrinsic mechanisms underlying the interaction between NDV and infected cells remain unclear.

Metabolomics, or global metabolic profiling, can be used to identify the special small metabolites of diseases in cancer and other diseases [4,5]. These metabolites may help researchers identify biomarkers for diagnosis and improve therapy through the design of target modulation drugs [6,7]. Metabolomics has already been widely used in biological studies of humans, animals, and plants [8,9]. Viruses are obligate parasites that hijack host cellular metabolism, limiting their growth and proliferation [10]. Recently, there has been increasing interest in exploring the effects of viral infection on host cellular metabolism. Many viruses have been proven to alter the metabolism in infected cells, including the human cytomegalovirus (HCMV), herpes virus, and human immunodeficiency virus [11,12,13,14]. Understanding the alterations of the virus in host cell metabolism will promote the knowledge of viral pathogenic mechanisms and contribute to advancing novel preventative measures.

Few studies have been performed on the host metabolism of NDV. Sun et al. established that there was significantly more histamine produced in the proventriculuses of infected birds than in the uninfected group [15]. The effects of NDV infection on lipid metabolism in black-bone chickens have also been determined [16]. Moreover, disturbing cholesterol homeostasis can also inhibit NDV replication [17]. However, no systematic alterations in host cellular metabolites after vNDV infection have been reported.

In the present study, cells and SPF chicken lung samples were collected from vNDV strain Herts/33-infected groups and then subjected to metabolomic analysis using ultra-high-performance liquid chromatography/quadrupole time-of-flight tandem mass spectrometry (UHPLC-QTOF-MS). The uninfected cells and SPF chicken lung samples were used as the controls. The results showed that the metabolome obtained through multivariate statistical analysis can distinguish between Herts/33-infected and uninfected groups, both in vitro and in vivo. Many metabolic pathways and metabolites may be associated with replication and pathogenicity of NDV.

## 2. Materials and Methods

### 2.1. Cell Culture and Virus

DF-1 cells were obtained from the American Type Culture Collection (ATCC, Manassas, VA, USA) and cultured in Dulbecco’s modified Eagle medium (DMEM, GIBCO, Thermo Scientific, Waltham, MA, USA) containing 10% fetal bovine serum (FBS, Invitrogen, Thermo Scientific, Waltham, MA, USA) and 1% penicillin/streptomycin at 37 °C with 5% CO_2_. The NDV strain Herts/33, obtained from the China Institute of Veterinary Drug Control (Beijing, China), was propagated in SPF chicken embryos. The 50% tissue culture infected dose (TCID_50_) was detected as described previously [18].

### 2.2. Virus Infection

DF-1 cells were seeded in T75 flasks and cultured overnight. When the cells reached approximately 80% confluence, they were infected with Herts/33 at a multiplicity of infection (MOI) of 1 and incubated at 37 °C with 5% CO_2_ for 1 h. Then, the virus inoculum was removed by washing with phosphate-buffered saline (PBS) and incubated in DMEM supplemented with 2% FBS in 5% CO_2_ at 37 °C. DF-1 cell samples were collected at 0, 6, 12, 18, and 24 h post-infection (h.p.i.). There were approximately 1 × 10^7^ cells per sample.

### 2.3. Sample Collection

For identification of the NDV infection, cell culture supernatants were collected for virus titration, and cells were lysed for Western blot analysis as described previously [19]. Briefly, cells were washed thoroughly and lysed in cell lysis buffer (P0013B, Beyotime, Shanghai, China). The lysates were denatured and then subjected to SDS-PAGE and transferred to nitrocellulose membranes (Whatman, Maidstone, UK). The membranes were then blocked and reacted with primary antibodies overnight at 4 °C and horseradish peroxidase (HRP)-conjugated secondary antibodies for 1 h at room temperature. The antibody–antigen complex was visualized using an enhanced chemiluminescence reagent solution kit (Share-bio Biotechnology, Shanghai, China) using a multi-chemiluminescence image analysis system (Tanon 5200, Tanon, Guangzhou, China). For metabolomic analysis, the cells were collected and washed quickly with ice-cold phosphate-buffered saline (PBS, pH 7.4). The cells were then digested with trypsin, and growth medium was added to stop digestion. The samples were then centrifuged to resuspend the cells in PBS. The cells were rapidly quenched by adding fivefold volumes of ice-cold quenching solution (60% aqueous methanol, 0.85% (*w*/*v*) ammonium bicarbonate, pH 7.4) [20]. The sample was centrifuged to remove the supernatant and the pellet was frozen by immersion in liquid nitrogen. Finally, samples were stored at −80 °C until metabolomic analysis.

### 2.4. Animal Experiments

One-day-old SPF chickens were obtained from Merial (Merial Vital Laboratory Animal Technology Company, Beijing, China) and kept at a controlled temperature (28–30 °C). The care and maintenance of all animals was performed in accordance with the Institutional Animal Care and Use Committee (IACUS) guidelines. The approval number is SHVRI-chicken-2018080408. Twelve 7-week -old SPF chickens were randomly divided into four groups of three. Chickens in one group were treated with PBS as the controls, and the chickens in the other three groups were treated with Herts/33 infection via nose and eye droppings for sample collection at 12, 24, and 48 h.p.i., respectively. Chicken lung samples (the right lower lobe of the lung) were collected and frozen by immersion in liquid nitrogen and were stored at −80 °C. For the metabolomic analysis, 50 mg of sample was taken for the metabolite extraction. The NDV infection in the lungs was confirmed by histopathological observation as described in previous works [21].

### 2.5. Metabolite Extraction

The samples were freeze-dried; then, 250 μL sterile water was added to the Eppendorf tubes and mixed by vortexing for 30 s. After adding magnetic beads, the samples were homogenized using a ball mill (JXFSTPRP-24, Jingxin Tech., Shanghai, China) at 45 Hz for 4 min. Then, they underwent ultrasound treatment for 5 min in ice water. The sample was homogenized three times, then centrifuged at 12,000 rpm for 15 min at 4 °C. The supernatant (200 μL) was extracted with 1000 μL of methanol/acetonitrile (1:1, *v*/*v*) containing ribitol (5 μg/mL) as an internal standard, followed by vortexing for 30 s. After ultrasound treatment for 10 min, the samples were incubated at −20 °C for 1 h, then centrifuged at 12,000 rpm at 4 °C for 15 min. The supernatant (825 μL) was transferred to a new Eppendorf tube, and the extracts were dried in a vacuum concentrator. The vacuum-dried extracts were dissolved with 100 μL of acetonitrile/water (1:1, *v*/*v*) and vortexed for 30 s, followed by ultrasound treatment for 10 min in ice water. The samples were centrifuged again at 12,000 rpm at 4 °C for 15 min. Finally, the supernatant (60 μL) was placed into a fresh 2 mL LC/MS glass vial. Approximately 10 μL was taken and mixed as a QC sample for the UHPLC-QTOF-MS detection.

### 2.6. LC–MS/MS Analysis

LC–MS/MS analyses were performed using an UHPLC system (Infinity 1290, Agilent Tech., Santa Clara, CA, USA) with a UPLC BEH Amide column (1.7 μm × 2.1 × 100 mm, Waters) coupled to an AB 6600 Triple TOF Mass Spectrometer (Q-TOF, AB Sciex, Concord, ON, Canada). The mobile phase consisted of two solutions: (A) 25 mM NH_4_OAc and 25 mM NH_4_OH in water (pH = 9.75); (B) acetonitrile. The gradient elution was as follows: 5:95 A/B at 0 min; 5:95 A/B at 0.5 min; 35:65 A/B at 7 min; 60:40 A/B at 8 min; and 60:40 A/B at 9 min; 5:95 A/B at 9.1 min; 5:95 A/B at 12 min. The flow rate was maintained at 0.5 mL per minute. The injection volume was 2 μL.

The AB 6600 Triple TOF MS can acquire the MS spectra of the first and second levels using the software (Analyst TF 1.7, AB Sciex, Boston, MA, USA) based on an information-dependent acquisition (IDA). In each cycle, precursor ions with the strongest strength and greater than 100 were collected for fragmentation. Collision energy (CE) was 30 V (15 MS/MS with a production accumulation time of 50 ms^−1^). The electrospray ionization (ESI) worked in positive or negative ion modes; the parameters were set as follows: Ion source gas 1, 60 Psi; ion source gas 2, 60 Psi; curtain gas, 35 Psi; source temperature, 600 °C; Ion Spray Voltage Floating range −4000 to 5000 V.

### 2.7. Statistical Analysis

We processed the original data to facilitate better analysis. MS raw data (.wiff) files were converted to the mzXML format using ProteoWizard software. Missing values in the original data were re-encoded and filled by the half of the minimum value method. The data were processed by R package XCMS (version 3.2, http://bioconductor.org/packages/release/bioc/html/xcms.html). In addition, the internal standard (IS) normalization method was used in this data analysis. The data obtained were used to search for the material information of all metabolites in the local database. The resulting three-dimensional data include the peak number, sample name, and normalized peak area, which were run through a series of multivariate pattern recognition analyses. SIMCA14.1 software package (V14.1, Sartorius Stedim Data Analytics AB, Umea, Sweden) was used for principal component analysis (PCA) and orthogonal projections to latent structures discriminant analysis (OPLS-DA) [22,23]. The analysis showed the distribution of origin data and classification of variables. To improve the analysis, the first principal component of variable importance in the projection (VIP > 1) and Student’s *t*-test (*p* < 0.05) were used to assess metabolites. The differential metabolites were identified according to the KEGG Metabolome Database, and MetaboAnalyst (http://www.metaboanalyst.ca/) was utilized to search for the pathways of metabolites [24].

## 3. Results

### 3.1. Replication of NDV in DF-1 Cells

**Figure 1:**
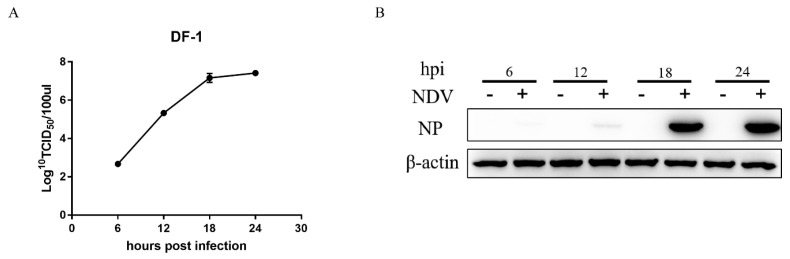
Detection of NDV infection in Herts/33-infected DF-1 cells. The cells were infected with NDV Herts/33 at 1 MOI, and samples were collected for detection at 6, 12, 18, and 24 h. (**A**) The NDV titers were determined using TCID_50_. (**B**) The NP protein of NDV was assessed by Western blot.

To confirm NDV replication in DF-1 cells, the cells were infected with a virulent Herts/33 strain at a MOI of 1, and the virus titer was determined. After infection with Herts/33, TCID_50_ was detected in infected-cell supernatant at 6, 12, 18, and 24 h.p.i. Virus titers in DF-1 cells progressively increased and reached a high level of approximately 10^7.5^ TCID_50_/0.1 mL at 24 h.p.i. (Figure 1A). In addition, the virus production was identified by measuring the expression level of NP using Western blot. As shown in Figure 1B, NP also increased over time. These results show that NDV could replicate effectively in DF-1 cells within 24 h of infection.

### 3.2. Multivariate Analysis of DF-1 Cell Metabolites

**Figure 2:**
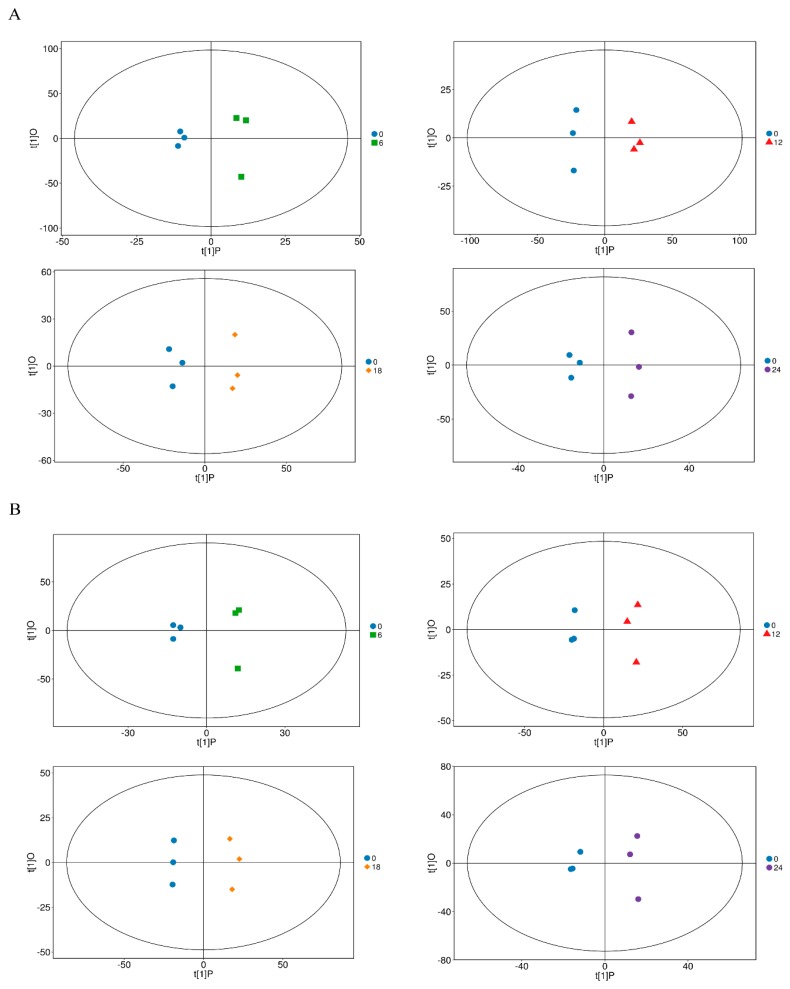
OPLS-DA model for the mock-infected and Herts/33-infected cells in different time courses. The OPLS-DA model (**A**,**B**) was derived from the UHPLC-QTOF-MS metabolomic profiles of the DF-1 cell samples. (**A**) was derived from POS and (**B**) from NEG.

Electrospray ionization served as the source of UHPLC-QTOF-MS, including positive and negative ion modes (POS and NEG). Based on an in-house MS2 database and the KEGG COMPOUND Metabolomics Library, the valid peaks were matched for 302 (POS) and 127 (NEG) DF-1 cell metabolites. To collect more reliable information about the intergroup differences of metabolites, we applied the OPLS-DA to analyze non-orthogonal variables and orthogonal variables. In this study, the OPLS-DA results for the mock and infected groups are shown in Figure 2A,B. The R^2^ and Q^2^ of the samples (POS, NEG) are shown in Table S1. The two groups were clearly distinguished; these results indicate that the infection model was reliable and stable.

### 3.3. Significant Differential Metabolites during NDV Infection

**Figure 3:**
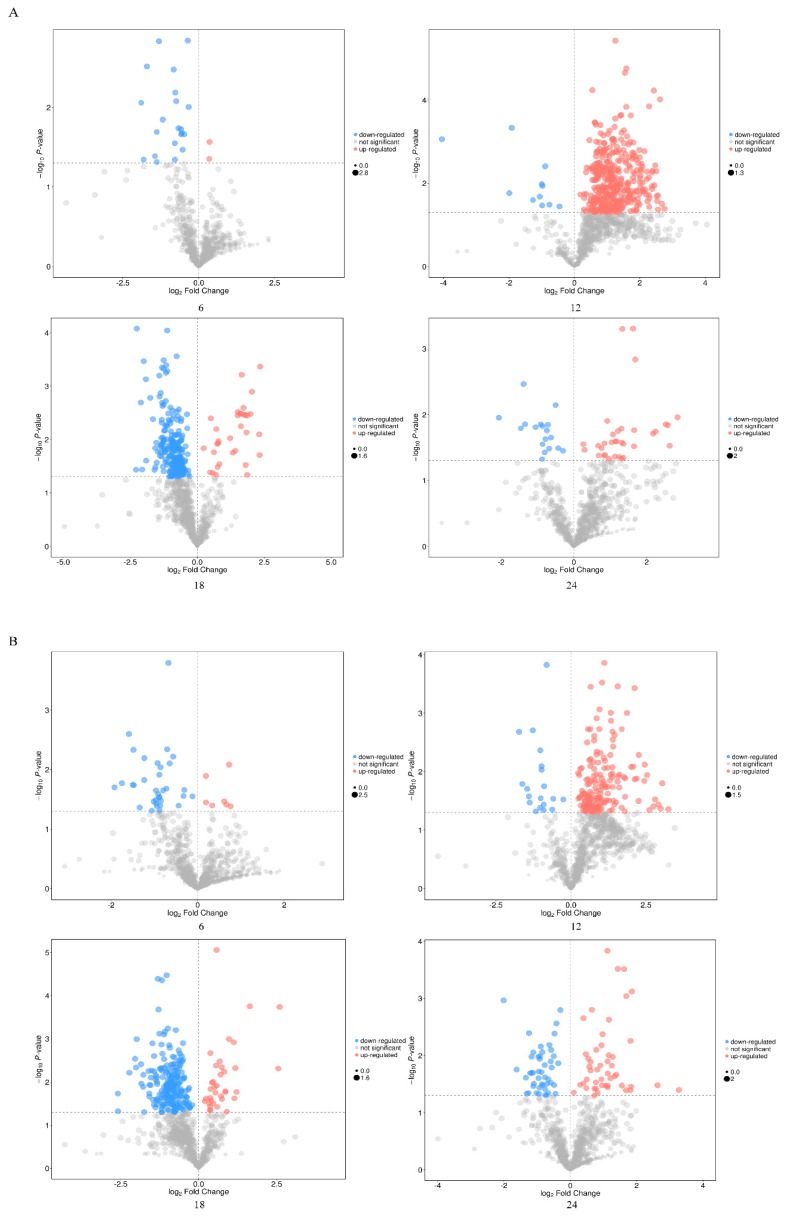
Volcano plots for the mock-infected and Herts/33-infected cells in different time courses. (**A**) was derived from POS and (**B**) from NEG. Each point in the volcanic map represents a metabolite. Red: Upregulation; blue: Downregulation; gray: Not significant.

To screen the differential metabolites, the VIP in the OPLS-DA model (VIP > 1) and *p*-value of Student’s *t*-test (*p* < 0.05) were used as the criteria. The results of screening differential metabolites were visualized in the form of volcano plots (Figure 3A,B). Each point in the volcanic map represents a metabolite.

**Figure 4:**
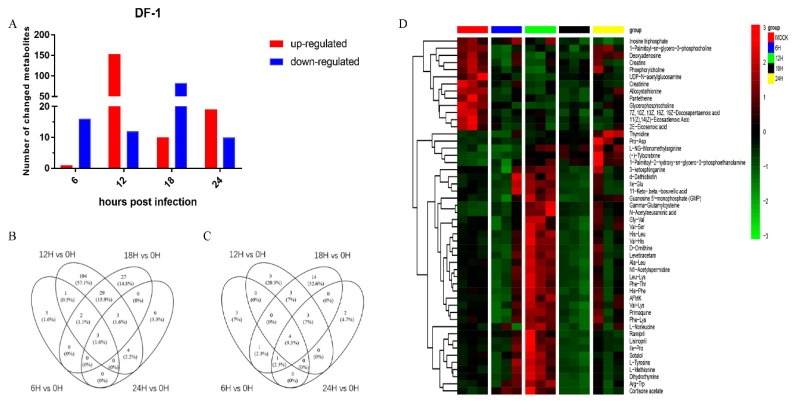
Analysis of differentially expressed metabolites in DF-1 cells infected with Herts/33 in different time courses. (**A**) Numbers of metabolites upregulated (red) and downregulated (blue) in infected cells. Venn diagrams (**B**,**C**) provide an overview of the global metabolite features in terms of their similarity and uniqueness for the five sampled groups. (**B**) was derived from POS and (**C**) from NEG. (**D**) Heatmap of hierarchical clustering analysis. Each column represents one sample, and each row represents one differential metabolite. The color of each cell represents the relative level of the differential metabolites. Red: Upregulation; green: Downregulation.

A total of 305 metabolites were significantly changed after Herts/33 infection, including 153 amino acids and their derivatives, 11 glycerophospholipids, 31 nucleotides and their derivatives, and others (Supplementary Materials Tables S2 and S3). Relative to values recorded at 0 h.p.i., 182 metabolites were significantly upregulated and 122 were significantly downregulated (Figure 4A). Venn diagrams (Figure 4B,C) provide an overview of the global metabolite features in terms of their similarity and uniqueness for the five groups. A significant difference was observed in the heatmap depicting hierarchical clustering of the metabolite data (Figure 4D). Metabolite variations were shown according to time post-infection. However, changes in upregulated metabolites were more abundant at 12 h.p.i. The results show that NDV infection caused a decrease in the levels of (4Z, 7Z, 10Z, 13Z, 16Z, 19Z)-Docosahexaenoic acid, 2E-Eicosenoic acid, and 11(Z), 14(Z)-Eicosadienoic acid, which are involved in the biosynthesis of unsaturated fatty acids. However, there was an increase in the levels of many amino acids, carboxylic acids and derivatives, and pyridines and derivatives, which are related to amino acid metabolism and nucleotide synthesis. The changes in these metabolites caused by NDV infection were found to contribute to viral replication.

### 3.4. Metabolic Pathway Analysis of Metabolites

**Figure 5:**
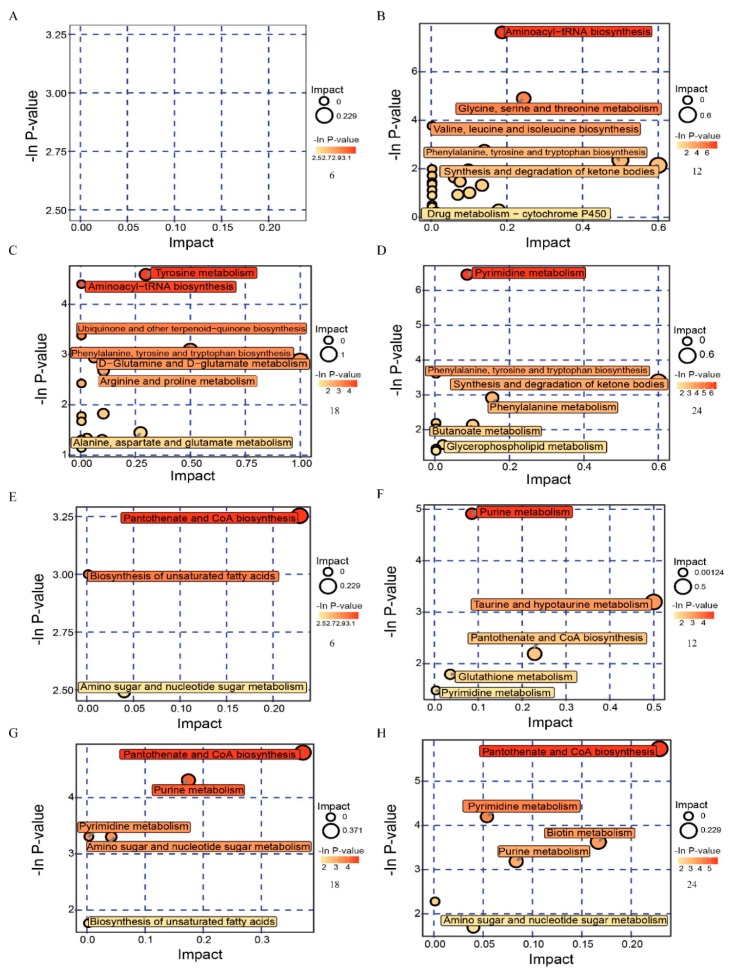
Bubble plots of the metabolic pathway analysis for DF-1 cells infected with Herts/33 in different time courses. (**A**–**D**) were derived from POS and (**E**–**H**) from NEG. Each bubble represents a metabolic pathway. The x-axis represents a pathway impact value in the topology analysis, and larger bubbles represent higher pathway impact values. The y-axis represents the *p*-value of the metabolic pathway in the enrichment analysis, and the darker color of the bubble represents higher pathway enrichment.

Differential metabolites were identified using the KEGG Metabolome Database and MetaboAnalyst. Next, we searched the corresponding pathways database of chicken, and further screened the pathways to identify those related to metabolite differences. The results of the metabolic pathway analysis are shown via a bubble plot (Figure 5). During the early stage of Herts/33 infection, few metabolic pathways were affected. Only three metabolic pathways were found to have changed at 6 h.p.i. (Figure 5A,E). They were pantothenate and CoA biosynthesis, the biosynthesis of unsaturated fatty acids, and amino sugar and nucleotide sugar metabolism. With the progress of virus replication, the numbers of the altered metabolic pathways increased. Pathway impact values indicate that the main enrichment metabolic pathways of differential metabolites after Herts/33 infection included aminoacyl-tRNA biosynthesis, phenylalanine, tyrosine and tryptophan biosynthesis, the synthesis and degradation of ketone bodies, purine metabolism, pyrimidine metabolism, amino sugar and nucleotide sugar metabolism, pantothenate and CoA biosynthesis, arginine and proline metabolism, alanine, aspartate, and glutamate metabolism.

**Figure 6:**
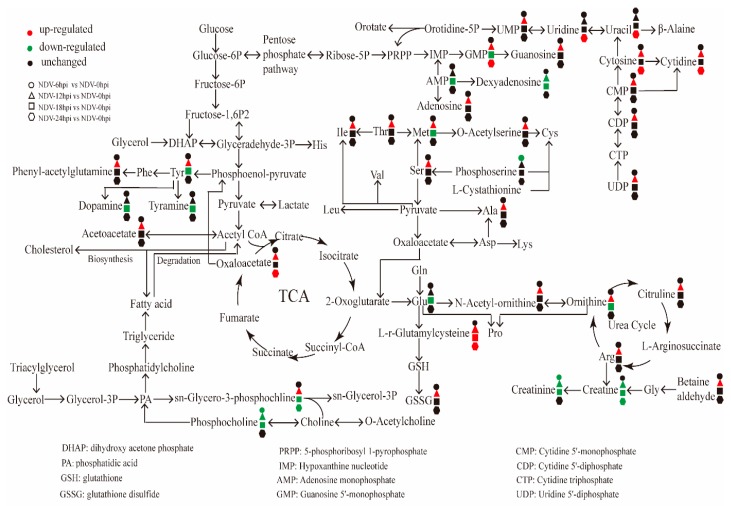
Schematic representation of altered metabolic pathways in DF-1 cells infected with Herts/33. Metabolomics of Herts/33-infected cells was performed by UHPLC-QTOF-MS, and many metabolites and associated metabolic pathways were significantly altered with the development of Herts/33, including amino acid metabolism, nucleotide metabolism, and urea cycles. Red: Upregulation; green: Downregulation.

The UHPLC-QTOF-MS results give us more detailed profiles of the metabolite changes in DF-1 cells infected with Herts/33 (Figure 6). As important substrates, amino acids are essential to cell metabolism and proliferation. In our results, tyrosine, isoleucine, threonine, methionine, serine, and alanine were upregulated, which allowed them to play an important role in many key metabolic pathways during Herts/33 infection. At 24 h.p.i., the levels of cytidine, cytosine, uracil, uridine, and GMP were the highest, which was consistent with the viral replication process. Further, creatinine, glycerophosphocholine, and sn-glycero-3-phosphocholine were downregulated during Herts/33 infection, the latter two of which were related to lipid metabolism.

### 3.5. Metabolic Changes Induced by Herts/33 Infection in Chicken Lungs

**Figure 7:**
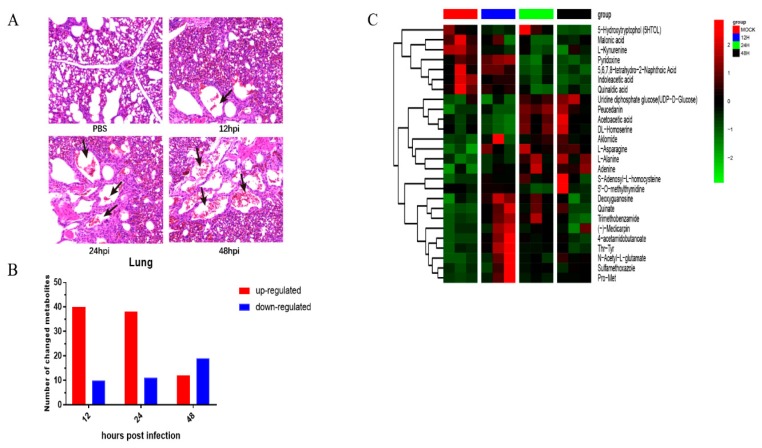
Analysis of differentially expressed metabolites in chickens infected with NDV Herts/33. (**A**) Hematoxylin and eosin staining of cells in a chicken lung (20 times magnification). Black arrows indicate lesions. (**B**) Numbers of metabolites upregulated (red) and downregulated (blue) in infected cells. (**C**) Heatmap of hierarchical clustering analysis. Each column represents one sample, and each row represents one differential metabolite. The color of each cell represents the relative level of the differential metabolite. Red: Upregulation; green: Downregulation.

An animal experiment was performed to investigate the effects of Herts/33 infection on lung metabolism in vivo. To confirm Herts/33 infection in the lungs, the lungs were subjected to histopathological observation with hematoxylin and eosin staining. There was local pulmonary hemorrhaging in the lungs of the Herts/33-infected group, but no pathological change was observed in the PBS group (Figure 7A). There were 130 metabolites that significantly changed after Herts/33 infection, including 31 amino acids and their derivatives, 6 carbohydrates, 8 glycerophospholipids, 14 nucleotides and their derivatives, and others (Supplementary Materials Tables S4 and S5). Compared with the control group, 90 metabolites were significantly upregulated and 30 were significantly downregulated (Figure 7B). A heatmap of differentially expressed metabolites in chickens infected with Herts/33 is shown in Figure 7C. The variation among metabolites was also related to different time points. Changes in upregulated metabolites were abundant at 12 and 24 h.p.i. There was also an increase in the levels of many amino acids, peptides, purines, and purine derivatives, which are related to amino acid metabolism and nucleotide synthesis. These results support that NDV infection could change the host metabolism for virus replication.

## 4. Discussion

In recent years, great achievements have been made in understanding of metabolic pathways and their contributions to viral genome replication, virion production, and survival in infected cells. Metabolomic analyses have been performed for some viruses, such as the human cytomegalovirus (HCMV), hepatitis C virus (HCV), and Zika virus [25,26,27]. Metabolic changes induced by viruses depend on the species of virus, and the metabolic perturbations are unique. In the present study, we analyzed the metabolomic profiles of Herts/33-infected DF-1 cells using UHPLC-QTOF-MS. In this way, this work provides new viewpoints on infected cells’ response to Herts/33 infection and interactions between the virus and host, which may help to determine the infection mechanism of vNDV.

This study provides more information on the alteration of metabolites and associated pathways in DF-1 cells after NDV infection. The results of OPLS-DA and hierarchical clustering revealed significant differences in the global metabolite profiles of controls and DF-1 cells infected with the NDV Herts/33 strain. There were significant changes in amino acids, lipid metabolism, purine metabolism, pyrimidine metabolism, and nitrogen metabolism (Figure 6). In a sense, the changes in these metabolites can reflect the intracellular reactions’ contributions to the understanding of how NDV regulates the host metabolic pathway to benefit virus proliferation.

The TCA cycle is known to play a vital role in in many cellular bioprocesses, which is also the hub of carbohydrate, lipid, and amino acid metabolism. However, the TCA cycle intermediates for ATP production were not significantly changed, except oxaloacetate during NDV infection. NDV has been reported to cause oxidative stresses and decrease the level of GSH and the activity of SOD, CAT, GST, GPx, and GR in the brain and liver of chickens [28,29]. Glutathione metabolism was affected (Figure 5) in Herts/33-infected DF-1 cells. Mitochondria, where oxidative phosphorylation takes place, products ROS as a by-product of ATP synthesis. Mitochondrial DNA injury may be caused by oxidative damage [30,31]. Thus, we surmised that mitochondrial DNA injury may be an effect of NDV-induced oxidative stress, which impairs the oxidative phosphorylation process.

Nitric oxide (NO) is an important physiologic messenger in vertebrates. It has various physiological functions [32,33]. Nitric oxide is made by nitric oxide synthase (NOS) in a reaction that converts arginine and oxygen into citrulline and NO. Many studies have demonstrated an increased level of NO in NDV-infected cells or chickens [34,35,36,37]. In our study, arginine metabolism was also increased (Figure 5 and Figure 6). Various metabolites involved in the urea cycle were changed. At 12 h.p.i., the rapid replication period of NDV, the levels of arginine, citrulline, ornithine, and N-Acetyl-ornithine were significantly increased. From another point of view, the higher level of NO caused by Herts/33 infection may be related to an increase in arginine metabolism.

Amino acids are some of the most important substances in organisms. They are not only utilized in the synthesis of proteins and other important biomolecules, but also provide intermediate metabolites for the tricarboxylic acid cycle and gluconeogenesis. The increase in amino acid metabolism caused by virus infection has been identified by many metabolomic studies [38,39,40,41]. Amino acid metabolism generally increased in KSHV latently-infected cells, such as large amounts of amino acids and metabolites related to their anabolic pathways [38]. In the present study, we obtained similar results. Amino acid metabolism was also upregulated, and the production of tyrosine, isoleucine, threonine, methionine, serine, and alanine also increased during Herts/33 infection. Similarly, the results of our in vivo experiments confirmed that amino acid metabolism was upregulated in the Herts/33-infected chicken lungs. The increased pools of amino acids may contribute to the rapid proliferation of viral protein synthesis and virion assembly.

Among the various metabolic pathways altered after Herts/33 infection, the changes in purine metabolism and pyrimidine metabolism are significant. Previous studies have shown that viruses satisfied the demand of viral nucleic acid synthesis by regulating the nucleotide anabolism, including the de novo synthesis pathway or salvage synthesis pathway [42,43]. Different viruses choose different ways to deal with the problem. NDV solved this problem by increasing the concentrations of the intermediates of purine and pyrimidine biosynthesis pathways. The levels of UDP, CMP, cytosine, uracil, UMP, and GMP also increased. The alterations in nucleotide metabolism may play an important role in facilitating the rapid viral genome replication of NDV.

## 5. Conclusions

In conclusion, the metabolome profiles of DF-1 cells infected with Herts/33 were analyzed to establish the metabolic characteristics by UHPLC-QTOF-MS. There were significant differences in amino acids, lipid metabolism, purine metabolism, pyrimidine metabolism, and nitrogen metabolism between the two groups. To obtain specific substrates and increase virion production, it is necessary for NDV to alter host cell metabolism. Similar results were also confirmed in vivo. Moreover, alterations in cellular metabolism may contribute to the survival of NDV-infected cells. The identification of these different metabolites and metabolic pathways will provide considerable important information for further understanding of NDV replication needs and pathogenesis.

## Supplementary Materials

Supplementary materials can be found at https://www.mdpi.com/1999-4915/11/10/962/s1. Table S1: R^2^ and Q^2^ of OPLS-DA model between the groups; Table S2. Identification and characterization of the metabolites in DF-1 cells infected with Herts/33 in POS mode; Table S3. Identification and characterization of the metabolites in DF-1 cells infected with Herts/33 in NEG mode; Table S4. Identification and characterization of the metabolites in chickens infected with Herts/33 in POS mode; Table S5. Identification and characterization of the metabolites in chickens infected with Herts/33 in NEG mode.
